# *Streptococcus agalactiae* Meningitis in Adult Patient: A Case Report and Literature Review

**DOI:** 10.1155/2016/6183602

**Published:** 2016-01-21

**Authors:** Fahmi Yousef Khan

**Affiliations:** Department of Medicine, Hamad General Hospital, Doha, Qatar

## Abstract

We report a case of group B streptococcus meningitis in a 72-year-old female patient who was admitted in our hospital with a 21-day history of bilateral lower thigh pain and swelling associated with fever, headache, and vomiting. Her past medical history was remarkable for DM type 2, hypertension, and hypothyroidism. Upon admission, examination showed bilateral warmth and tender soft tissue swelling around the knees and MRI showed cellulitis of distal thirds of both thighs. The next day, the patient became drowsy. Neurologic examination showed neck rigidity and right sided hemiparesis. Cerebrospinal fluid and blood cultures yielded group B streptococcus sensitive to ceftriaxone, penicillin G, and vancomycin. The patient received ceftriaxone for a total of 14 days after which she improved and was discharged from the hospital with right sided weakness.

## 1. Introduction

Meningitis caused by GBS predominates in children younger than 3 years as a result of the mother's transmission of contamination from the childbirth channel. Although it was considered rare in the past [1], GBS meningitis in adults has become increasingly frequent in recent years [2]. In our hospital, review of microbiology laboratory records from 2006 to 2012 identified four cases of GBS meningitis. There were three neonates and one adult. In this report, we added one case of GBS meningitis in nonpregnant women to the previously described ones in the literature [2–7], to increase physicians' awareness of this disease among adult patients.

## 2. Case History

A 72-year-old female patient presented to the emergency department with 21-day history of progressively worsening bilateral lower thigh pain and swelling associated with fever, headache, and vomiting. Her past medical history was remarkable for DM type 2, hypertension, and hypothyroidism.

On examination, the patient was sick and tachypneic. Blood pressure was 100/50 mmHg, pulse was 110/min, body temperature was 39.8°C, and respiratory rate was 30/min. Lower limbs examination showed bilateral warmth and tender soft tissue swelling around the knees. She was unable to flex or extend her knees completely. The rest of the examination was unremarkable.

Laboratory examinations revealed a white blood cell count of 15,100/*μ*L with 70% neutrophils and 23% lymphocytes. Results of urinalysis were unremarkable. MRI of both knees showed bilateral osteoarthritis and effusion with cellulitis of distal thirds of both thighs. MRI showed subcutaneous edema of distal third of both thighs and bilateral knee osteoarthritis. Blood samples were submitted for cultures and patient commenced on intravenous piperacillin/Tazobactam.

The next day, the patient became drowsy. Neurologic examination showed neck rigidity and right sided hemiparesis. The cerebrospinal fluid (CSF) was turbid with 10,000 leukocytes/*μ*L. The Gram stained smear of CSF showed Gram-positive cocci in chains, with a protein concentration of 164 mg/dL and a glucose concentration of 21 mg/dL. A diagnosis of bacterial meningitis was made and piperacillin/Tazobactam was stopped, while the patient was empirically treated with intravenous ceftriaxone (4 g/day) and vancomycin (2 g/day). Brain CT showed multiple focal densities seen at basal ganglia region and left internal capsule suggestive of lacunar infarcts. On the following days CSF and blood cultures yielded* Streptococci agalactiae* or group B streptococcus (GBS) sensitive to ceftriaxone, penicillin G, and vancomycin. A transthoracic echocardiogram was negative for endocarditis. Ceftriaxone was continued for a total of 14 days after which the patient improved and was discharged with right sided weakness.

## 3. Discussion

GPS are the leading cause of puerperal infections and sepsis, as well as neonatal meningitis [8]. In adults, GBS meningitis is relatively rare representing between 0.3% and 4.3% of all cases of bacterial meningitis; most of them have been community acquired [2, 8]. In our hospital GBS meningitis accounts for 0.5% of total adult cases of bacterial meningitis, which falls within the above-mentioned range.

Adult GBS meningitis usually occurs in patients with underlying conditions [9–11] or in pregnancy and puerperium [12–15]. However, some patients might not have predisposing factors [3–6]. In some of these cases, an obvious source of infection commonly involving the endometrium, respiratory tract, and endocardium can be identified [7]. Domingo et al. [2] revealed that 86 per cent of the patients had comorbid conditions and 50 per cent had a distant focus of infection. Our patient was diabetic and she got the infection most probably secondary to bacteremia, with thigh cellulitis being the source of infection.

Clinical picture of adult GBS meningitis and CSF findings are indistinguishable from other bacterial meningitis. Our patient presented with fever, headache, vomiting, consciousness disturbance, and neck rigidity, which are nonspecific and can be found in other bacterial meningitis [16, 17].

Although group B streptococci are 4- to 10-fold less susceptible to penicillin than group A streptococci [15], penicillin is still the first choice of treatment and third-generation cephalosporins, especially ceftriaxone, were suitable alternative agent. Our patient showed dramatic response to ceftriaxone.

The mortality rate was 34 per cent [2]. Factors associated with high mortality include advanced age, neurological complications such as coma or focal neurological signs, and/or extraneurological complications such as shock, acute respiratory failure, acute renal failure, or consumption coagulopathy [2]. However, the outcome in our patient was favorable despite advanced age and neurological complications.

In conclusion, although GBS meningitis is an unusual manifestation of invasive GBS disease in adults, clinicians should be aware of this clinical entity and that it usually occurs in patients with underlying conditions and usually associated with a source of infection.
